# Rectal cancer MRI: protocols, signs and future perspectives radiologists should consider in everyday clinical practice

**DOI:** 10.1007/s13244-018-0606-5

**Published:** 2018-04-19

**Authors:** Andrea Delli Pizzi, Raffaella Basilico, Roberta Cianci, Barbara Seccia, Mauro Timpani, Alessandra Tavoletta, Daniele Caposiena, Barbara Faricelli, Daniela Gabrielli, Massimo Caulo

**Affiliations:** 1 ITAB Institute of Advanced Biomedical Technologies, University “G. d’Annunzio”, Via Luigi Polacchi, 11 66100 Chieti, Italy; 2 SS Annunziata Hospital, Department of Neuroscience, Imaging and Clinical Sciences, University “G. d’Annunzio”, 66100 Chieti, Italy

**Keywords:** Magnetic resonance imaging, Diffusion-weighted imaging, Locally advanced rectal cancer, Chemo-radiation treatment response, Complete responders

## Abstract

**Abstract:**

Magnetic resonance imaging (MRI) allows to non-invasively evaluate rectal cancer staging and to assess the presence of “prognostic signs” such as the distance from the anorectal junction, the mesorectal fascia infiltration and the extramural vascular invasion. Moreover, MRI plays a crucial role in the assessment of treatment response after chemo-radiation therapy, especially considering the growing interest in the new conservative policy (wait and see, minimally invasive surgery). We present a practical overview regarding the state of the art of the MRI protocol, the main signs that radiologists should consider for their reports during their clinical activity and future perspectives.

**Teaching Points:**

• *MRI protocol for rectal cancer staging and re-staging.*

• *MRI findings that radiologists should consider for reports during everyday clinical activity.*

• *Perspectives regarding the development of latest technologies.*

## Introduction

Magnetic resonance imaging (MRI) represents the first-choice exam for rectal cancer staging. Usually, clinicians require rectal MRI in case of positive colonoscopy for cancer and/or impossibility to endoscopically remove the lesion. In this context, the role of other imaging modalities, such as endorectal ultrasound and computed tomography, has some limitations. Endorectal ultrasound has the advantage, compared to MRI, of being able to differentiate T1-T2 tumours. However, in case of T3-T4 tumours, it is burdened by its limited field of view, which negatively impacts on the assessment of the mesorectal fat involvement and of tumour extension. Moreover, it is a highly operator-dependent method, requiring a not straightforward learning curve to reach an optimal diagnostic accuracy [1]. Computed tomography offers the advantage of a panoramic view of the rectum with the possibility to benefit from multiplanar reconstructions. It provides appreciable values of accuracy in the staging of high- and middle-rectum cancers [positive predictive value (PPV) and negative predictive value (NPV) of 86% and 94% respectively] but suffers from the low contrast resolution if the tumour is located in the low rectum (PPV and NPV of 53% and 73% respectively) [2].

For these reasons and thanks to its high contrast resolution, MRI allows to non-invasively evaluate the tumour site and, more generally, to obtain a highly accurate rectal cancer staging, which is essential to decide the appropriate treatment strategy. Furthermore, MRI allows the accurate detection of “prognostic signs” such as the distance between the caudal tumour margin and the anorectal junction, the mesorectal fascia infiltration and the presence of extramural vascular invasion.

Finally, in the light of the growing interest in the new conservative policy (wait and see, minimally invasive surgery), the availability of advanced MRI techniques, such as diffusion-weighted imaging (DWI) and dynamic contrast-enhanced (DCE) imaging, offers new perspectives concerning the assessment of treatment response.

Recently, the 2012 European Society of Gastrointestinal and Abdominal Radiology consensus guidelines were updated, providing new recommendations concerning the acquisition, interpretation and reporting of MRI for clinical staging and restaging of rectal cancer (Table 1) [3].

**Table 1:** Main updated imaging interpretation and reporting recommendations by 2016 ESGAR consensus meeting

• T3 and T4 substages should be reported (T3a, b, c, d and T4a, b)
• Structured report including the circumferential location of the tumour
• Nodal staging and restaging (according to short axis and morphological criteria concerning shape, border and signal)
• Presence of EMVI (increased risk of tumour recurrence and impaired overall survival)
• Sphincter invasion, involvement of MRF, pelvic wall/floor (T4), peritoneal reflection (T4)

The aim of this review is to provide a practical overview (summarised in Table 2) regarding the state of the art of the MRI protocol for rectal cancer pointing out the signs that radiologists should consider for their reports during every day clinical activity. Additionally, future perspectives regarding the development of the latest technologies that could be implemented in the near future will be further discussed.

**Table 2:** MRI protocol and diagnostic elements for rectal cancer that radiologists should consider in their clinical activity and report

MR Protocol	MRI Staging	MRI Restaging
✓ T2-weighted axial perpendicular to the main tumour axis (slice thickness ≤3 mm) ✓ DWI with the same T2-weighted inclination ✓ high *b* value (≥800 mm^2^/s) ✓ endorectal filling and anti-peristaltic drugs in selected cases	✓ site (low, middle, high rectum) of the tumour (circumferential location) ✓ longitudinal extension of the tumour ✓ distance of the caudal tumour margin from anorectal junction ✓ mesorectal involvement and tumour free resection margin ✓ extramural vascular invasion (EMVI) ✓ nodal involvement (size, shape, MR signal and borders)	✓ same criteria used for the MR staging regarding T ✓ specified criteria concerning nodal restaging ✓ potential pitfalls on DWI (misinterpretation of low signal on ADC map from fibrosis, susceptibility effects, T2 shine-through of fluid in rectal lumen, suboptimal sequence angulation and collapsed rectal wall)

## MRI protocol

In recent years the availability of powerful MRI scanners, operating at field strengths of 3 T, has attracted the interest of researchers regarding the possibility to improve image quality and facilitate clinical decisions. In this regard, a study by Maas et al. [4] compared the accuracy of 3-T and 1.5-T scanners to discriminate between T2 and borderline T3 rectal cancers when performing exams on the same group of patients. The expectation was that the use of a 3-T scanner would be beneficial because of its higher resolution due to the higher signal/noise ratio. However, results revealed no significant differences between the two MRI scanners. The reason was that the same difficulties related to the interpretation of desmoplastic reaction, with or without tumour cells, encountered with a 1.5-T scanner were confirmed using a 3-T scanner.

Among types of coils, currently the phase array external coils represent the state of the art in rectal cancer imaging. Endorectal coils, although offering better visualisation of the rectal wall and providing similar performances to endorectal ultrasound, are burdened with a poor patient compliance, especially in the case of stenosing tumours. For these reasons, their use is currently considered obsolete [5].

Some studies were recently published regarding the usefulness of endorectal contrast or filling during the MRI exam. Basically, there are two schools of thought and the potential advantage of the use of endorectal gel would be twofold. Firstly, the distention of lumen by means of endorectal gel should provide an optimal delineation of small (<3 cm) tumours or polypoid lesions [6]. Secondly, DWI should be beneficial for the reduction of intra-luminal air with subsequent decrease of susceptibility artefacts [7]. On the other hand, some studies underline the possibility that distension of the lumen and the subsequent compression on the mesorectum may hamper the detection of perirectal lymph nodes and lead to overestimation of the mesorectal fascia infiltration [8]. To date, the routine administration of endorectal filling is not recommended [3]. In the same way, the use of antiperistaltic drugs should be reserved to specific cases (for example, when the tumour is in the high rectum) to reduce the motion artefacts and it is not routinely performed [3].

Each rectal MRI protocol should include T2-weighted sequences and DWI. Slice thickness of 3 mm or less is considered a good compromise in terms of contrast and spatial resolution between tumour and soft tissue, mesorectal fat and mesorectal fascia [3]. Thicker slices might lead to losing small nodes and information regarding the distance between tumour and mesorectal fascia.

T2-weighted axial images should be preferentially acquired in high resolution and perpendicularly to the major axis of the tumour because this technique allows to better visualise the tumour and to correctly assess the distance between tumour and mesorectal fascia. In the same way, DWI should be performed with the same angulation of T2-weighted images, used as a reference and include high *b* values (≥800).

## Primary staging

### T staging

The site (high, middle and low rectum), the morphology, the tumour length and the distance of the caudal margin of the tumour from the anorectal junction should be described. Concerning the site, the circumferential location of the tumour should be considered. Regarding the distance from the anorectal junction, it is essential to provide the surgeons with information as detailed as possible regarding the distal margin and the invasion of the anal sphincter muscles. Slater et al. [8] recommended a distal margin of 1 cm and 2 cm for an oncologically safe resection in T1-T2 and T3-T4 tumours respectively. However, more recent studies demonstrated that these criteria are nowadays questionable and widely influenced by the local experience of the surgeon and his policy [10–12]. Zeng et al. [13] recently investigated the prognostic value of a distal margin ≤1 mm after sphincter-preserving resection for rectal cancer. Compared with negative distal margin (>1 mm), these patients showed higher 5-year local recurrence rate (24.1% vs 12.0%) and inferior 5-year disease-free survival (45.5% vs 69.5%). Interestingly, among patients with positive distal margin, those who received adjuvant therapy demonstrated higher 5-year disease-free survival (52.0% vs 30.7%). Furthermore, MRI allows to correctly identify the mesorectal fat infiltration, which is suggestive for T3 staging, the presence of adjacent organ infiltration (T4) and pathological nodes. In this context, the detection of locally advanced rectal cancer (T3 cd/T4, N2, M0) is crucial because these patients show a high rate of local recurrence (approximately 25%) if not treated with a neoadjuvant approach. At the same time, MRI allows the preoperative identification of good prognosis tumours (T3ab, N0) that have, similarly to T2 N0 tumours, a good outcome with primary surgery alone. Concerning T3abN1 tumours, they are currently not defined under “high risk” and can be treated with surgery alone as well [14, 15]. Moreover, the distance between tumour and mesorectal fascia (tumour-free resection margin) and the location of the shortest distance between tumour and mesorectal fascia should always be assessed because it represents an important prognostic factor. In particular, the presence of tumour within 1 mm of the surgical circumferential resection margins is an independent predictor of survival and local recurrence [15].

Particular attention should be used in the assessment of the relationship between tumour and peritoneal reflection onto the anterior aspect of the rectum, which is more appreciable viewing sagittal T2 images: the peritoneal infiltration changes the T stage from T3 to T4a [16].

In the light of the last recommendations of the 2016 ESGAR consensus, radiologists, regardless of the TNM, should provide specific information concerning the involvement of the internal sphincter, the inter-sphincteric plane and the external sphincter. Furthermore, they should indicate if the upper third, middle third or the lower third of the sphincter complex is involved. The involvement of the pelvic wall (levator ani muscle) should be considered as T4 [3].

### N staging

A complete report should include the assessment of perirectal nodes in the mesorectum as well as nodes along the superior rectal vessels and lateral nodes along internal iliac vessels. The presence of pathological nodes in these sites is related to the development of local or distant metastasis, and for this reason, they should be included in the radiation field. Currently there are no conclusive criteria to differentiate normal and pathological nodes with reasonably high sensitivity and specificity values. The accuracy of MRI in the primary nodal staging ranges from 55 to 78% using the size criteria alone [17, 18]. This happens because also very small nodes (<5 mm) can be metastatic. However, recent studies recommend to decide about their potential malignancy on the basis not only of their small diameter but also considering their morphology (round versus oval), signal intensity (homogeneous signal with adipose hilum preserved versus inhomogeneous signal with loss of adipose hilum) and borders (regular versus irregular). In this way, accuracy of MRI improves results in 85% and 97% in terms of sensitivity and specificity respectively [19]. Nevertheless, the correct classification of small nodes (<4 mm) is complicated, and the debate in the literature is open to explore other possibilities. Lambregts et al. [20] investigated the feasibility of the use of Gadofosveset for nodal staging and restaging. In their study, the use of nodal signal intensity and the “chemical shift” artefact were proven to be beneficial for the detection of malignant nodes, improving the area under the receiver operator characteristic curve (AUC) from 0.84 of the standard MRI to 0.96.

Specified criteria for nodal staging emerged from 2016 ESGAR consensus [3]. Basically, they recommend to assess nodes according to dimensional and morphological criteria. In detail, nodes with a short axis greater than 9 mm and all mucinous nodes should be considered malignant regardless of morphological criteria. Concerning nodes with a short axis between 5 mm and 9 mm or less than 5 mm, the final decision should be taken according to the morphological criteria (round shape, irregular border and heterogeneous signal).

Extramural vascular invasion (EMVI) refers to the presence of tumour cells within the veins outside the bowel wall and, if present, should be always included in the report. Although the presence of EMVI does not change the TNM staging, it is related to an increased risk of tumour recurrence and impaired overall survival and should be reported by radiologists. It should be detected as an irregularity or a nodularity of the vein contour in the vicinity of the tumour [21].

## Restaging after chemoradiation therapy before surgery

Locally advanced rectal cancers are usually treated with neo-adjuvant chemoradiation therapy. MR is often performed at the end of the treatment, usually after 6–8 weeks, before surgery, to assess the treatment response. In this context, radiologists are called upon to describe the entity of the tumour reduction, if present, in terms of longitudinal extension and of mesorectal involvement degree, and mostly, to recognise any residual tumour foci which would be suggestive of an incomplete response. This assessment represents a crucial step in the clinical history of patients. In light of recent studies, selected patients (with no residual tumour appreciable on restaging MR, negative endoscopy and negative digital exploration at the end of treatment) may avoid surgery and benefit from a wait-and-see policy [22].

In this regard, each restaging MRI report should include a radiologist’s opinion indicating if the patient is a non-responder, a partial responder or a complete responder. When the residual tumour is still detectable on the T2-weighted images, radiologists should assess, if present, the extent of the response, in terms of tumour size reduction. However, in most cases, the presence of radiation therapy-induced fibrosis makes the evaluation of treatment response difficult because it can hide small areas of residual vital tumour. For this reason, the accuracy of conventional MRI in the assessment of treatment response is quite low (around 50%) and it is even lower (19%) regarding the detection of complete responder patients [23].

DWI is a relatively new technique which investigates the motion of water molecules, allowing the non-invasive assessment of cellularity. On the one hand, the role of DWI in the primary staging is still debated in the literature; on the other hand, its importance is mainly underlined at the restaging after neoadjuvant treatment. Its effectiveness in the restaging after neoadjuvant treatment has been proven in several studies. In detail, Kim et al. [24] demonstrated a significant improvement of the MRI accuracy when adding DWI to conventional MRI, showing AUC value of 0.88.

The principle of DWI interpretation is relatively simple and is related to the detection or absence of hyperintense foci on the site of the primary tumour, which is suggestive respectively for residual tumour or a complete response. Despite of the clear usefulness of DWI, the interpretation of images requires a certain degree of experience because some pitfalls, such as misinterpretation of low signal on ADC map from fibrosis, susceptibility effects, T2 shine-through of fluid in the rectal lumen, suboptimal sequence angulation and collapsed rectal wall, may hamper the diagnostic performances of non-expert radiologists [7]. Among these, “T2 shine-through effect” can be described as hyperintense signal detected on DWI which is not due to an actual restricted diffusion, but is related to the intrinsic properties (long T2 decay time) of some normal tissues. In this context, the apparent diffusion coefficient (ADC) is useful to discriminate the artefact from an actual restricted diffusion revealing an hypointense signal in case of residual tumour and hyperintense signal corresponding to the artefact.

Mucinous tumours should be considered and evaluated independently from the DWI. In fact, their mucin content is responsible for high DWI values despite the treatment [25].

Some studies have investigated the usefulness of ADC maps, due to their quantitative nature, to assess their potential as a predictive biomarker of treatment response. Several studies revealed significant differences in terms of pre-treatment ADC between poor responder patients and good responders [26–28]. Considering the ADC variation between the pre-treatment and the pre-surgery exams, Genovesi et al. [29] revealed that a cut-off of 29.5% in terms of increment of ADC had a sensitivity of 83.3%, specificity of 90%, a PPV of 91% and an NPV of 82%.

Anyway, results currently available in the literature are inconsistent, and, for this reason, routine ADC quantification is still not recommended [24, 30–32]. Furthermore, the hypointensity on ADC map due to radiation-induced fibrosis can mimic a residual tumour with subsequent misinterpretation and increase of false-positive rate [31].

Regarding the nodal involvement, a comparison with the pre-treatment exam should be performed according to the same features described for the primary staging (diameter variations, signal intensity, morphology and margins) but recent studies underline the usefulness of size criteria. In detail, Heijnen et al. [33] revealed that a cut-off size of 2.5 mm was predictive of nodal metastasis with an AUC of 0.78, and a sensitivity and specificity of 75% and 64% respectively; additionally, a decrease in size ≥70% predicts ypN0 status with 100% of accuracy. More generally, recent studies show that nodal restaging after chemoradiation therapy is more accurate compared with the primary staging [34, 35]. There are two main explanations: firstly, the rate of malignant nodes is significantly lower at the restaging compared with the staging (36% versus 97%) with a subsequent reduction in terms of false negative findings; secondly, most of the smaller nodes tend to disappear after chemoradiation therapy. In this regard, a recent study of van Heeswijk et al. [36] suggested that the absence of appreciable nodes on restaging DWI is a reliable predictor of complete response to neoadjuvant treatment. In detail, results showed a negative predictive value of 100% in the discrimination between a yN0 and yN-positive; moreover, the absence of nodes was greatest in patients with a complete response.

In the light of the last recommendations, after a long course of neoadjuvant treatment, all nodes with a short axis <5 mm should be considered benign, while morphological criteria should be used for short diameter ≥5 mm nodes [3].

## Future perspectives

The availability of a new policy (wait and see) and new technologies [positron emission tomography/computed tomography (PET/CT), DWI and dynamic contrast-enhanced magnetic resonance imaging (DCE-MRI)] leads clinicians to be more demanding with radiologists than in the past, regarding the prediction of the tumour clinical history (aggressiveness, treatment response, local or distance recurrence) (Table 3). Fusco et al. [37] recently published a systematic review focusing on the role of several imaging modalities in diagnosis, staging and pre-surgery treatment response assessment in locally advanced rectal cancer (LARC). In detail, they concluded that traditional morphological MRI is the modality of choice for rectal cancer staging, allowing accurate assessment of the disease extent, lymph node involvement, mesorectal fascia and sphincter complex. On the other hand, the integration of these modalities, including DCE-MR, DWI and PET/CT showed high accuracy to evaluate preoperative therapy response (85% sensitivity and 96% specificity). Several studies investigated the potential predictive role of FDG PET/CT for the treatment response assessment in LARC [38, 39]. For example, Cascini et al. [40] demonstrated that the change in tumour ^18^F-fluorodeoxyglucose (18F–FDG) standardised uptake value (SUV) is an early predictor of pathological tumour response revealing variations change significantly lower in non-responder patients than in responders. In 2012, Avallone et al. [41] demonstrated that the early change (before and 12 days after the beginning of the chemoradiotherapy treatment) in tumour 18F–FDG SUV is a reliable pre-surgical predictor of tumour recurrence. A recent study by Pecori et al. [42] showed correlations between metabolic response and pathological tumour response in patients undergoing neoadjuvant short course radiotherapy with delayed surgery for LARC.

**Table 3:** Main topic of research and future perspectives

• Diffusion-weighted imaging (DWI, intravoxel incoherent motion)
• Dynamic contrast-enhanced magnetic resonance imaging
• Positron emission tomography/computed tomography (PET/CT)
• Imaging integration (PET/CT, DCE-MRI, DWI)
• Radiomics
• Deep learning and automatic segmentation tool

Concerning the treatment response prediction with DWI, in the light of good results obtained by using whole-volume tumour segmentations [32, 43, 44], a growing interest has developed in the literature to explore the feasibility of automatic segmentation tools. The main advantage of this approach would be that manual segmentation of the whole tumour is a time-consuming procedure during the everyday clinical practice, requiring up to 18 min [45] and high-level experience. Trebeschi et al. [46] used deep learning methods (convolutional neural networks) to obtain an automatic localisation and segmentation of rectal cancer on multiparametric MRI with promising results. In detail, in this study, the AUC of the probability maps resulting from the validation of the computational neural network classifier reached 0.99.

A potential limitation of DWI is that to date there are no studies showing a conclusive predictive role for the primary staging MRI exam. Concerning this research topic, some studies recently investigated the potential role of perfusion MRI (or DCE-MRI), which allows the analysis of tumour neo-angiogenesis, in terms of pharmacokinetic parameters, using an intravenously administered contrast agent (gadolinium-DTPA or gadolinium-DOTA). The thesis is that, at the primary staging, the angiogenesis of an aggressive tumour would be different from that of a non-aggressive tumour. Moreover, DCE should improve the conspicuity of small residual tumour foci at restaging MRI. For example, Oberholzer et al. [47], in a large prospective study, demonstrated that DCE curves and semi-quantitative perfusion parameters were able to distinguish between good responder patients and poor responders. More recently, in 2015, Petrillo et al. [48] investigated the predictive role of MRI volumetry based on signal intensity characteristics on DCE to distinguish between complete and incomplete responders. They showed that diagnostic performance linked to DCE-MRI volumetric change was superior to T2-weighted and DW-MRI volumetric change performance. In another study, the same group investigated several shape parameters for the time-intensity curve (TIC) in order to identify the best combination of predictive parameters to distinguish responders and non-responder patients. As a result, they proposed a standardised index of shape (SIS), which is a semi-quantitative angiogenic biomarker providing an estimation for tumour blood perfusion [49]. Furthermore, this approach revealed a higher predictive ability compared to the SUV (PET-TC) and to DWI parameters [50, 51]. Limits of this technique are related to field inhomogeneity induced on T1 and the need of high-field scanner, preferentially 3 T.

Perfusion MRI is not the only way researchers are following. Due to inconsistent results reported in literature regarding the prediction of treatment response by using ADC values, Nougaret et al. [52] recently described a new approach to analyse DWI data by using a bi-exponential function, called IVIM (intravoxel incoherent motion). In detail, two types of “diffusion contributes” would be responsible for the IVIM-derived diffusion coefficient D, the microcirculation or perfusion effects (pseudo-diffusion) and the true tissue diffusion. Both of these contributions can be estimated by means of a single diffusion imaging acquisition using low *b* value (<200 mm^2^/s) to quantify pseudo-diffusion and high *b* value (>200 mm^2^/s) to quantify the true tissue diffusion. In this study, they demonstrated the parameter D was useful to discriminate between good and poor responders.

Another exciting topic in the literature regards data analysis providing quantitative features extracted from images by means of a radiomics approach. Three orders of quantitative features can be extracted: first-order features are based on tumour shape, size, intensity and volume; second-order features are generally described as “texture features” and are related to voxel similarity (or dissimilarity) in terms of contrast values; third-order features describe the presence of repetitive or non-repetitive patterns of voxels [53]. All these features can be combined in large databases and correlated with clinical outcomes by means of a machine learning approach. The objective is to obtain imaging biomarkers suitable for the clinical outcome prediction.

## Conclusions

Magnetic resonance is considered the first-choice technique for the rectal cancer primary staging and restaging after neoadjuvant treatment. Recent recommendations by the European Society of Gastrointestinal and Abdominal Radiology help radiologists to improve their everyday clinical activity with regard to image acquisition, interpretation and reporting. Nowadays, the integration of different imaging modalities, new image analysis approaches and the research of predictive imaging biomarkers represent a promising perspective.
